# Identifying key elements for paediatric advance care planning with parents, healthcare providers and stakeholders: A qualitative study

**DOI:** 10.1177/0269216319900317

**Published:** 2020-01-27

**Authors:** Kerstin Hein, Kathrin Knochel, Vedrana Zaimovic, Daniel Reimann, Anna Monz, Nari Heitkamp, Gian Domenico Borasio, Monika Führer

**Affiliations:** 1 Center for Pediatric Palliative Care, University Children’s Hospital, Ludwig-Maximilians University of Munich, Munich, Germany; 2 Palliative Care Service, Centre Hospitalier Universitaire Vaudois, Lausanne, Switzerland

**Keywords:** Advance care planning, advance directives, decision-making, palliative care, paediatrics

## Abstract

**Background:**

Although international guidelines recommend discussions about goals of care and treatment options for children with severe and life-limiting conditions, there are still few structured models of paediatric advance care planning.

**Aim:**

The study aimed at identifying key components of paediatric advance care planning through direct discussions with all involved parties.

**Design:**

The study had a qualitative design with a participatory approach. Participants constituted an advisory board and took part in two transdisciplinary workshops. Data were collected in discussion and dialogue groups and analysed using content analysis.

**Setting/participants:**

We included bereaved parents, health care providers and stakeholders of care networks.

**Results:**

Key elements were discussions, documentation, implementation, timing and participation of children and adolescents. Parents engage in discussions with facilitators and persons of trust to reach a decision. Documentation constitutes the focus of professionals, who endorse brief recommendations for procedures in case of emergencies, supplemented by larger advance directives. Implementation hindrances include emotional barriers of stakeholders, disagreements between parents and professionals and difficulties with emergency services. Discussion timing should take into account parental readiness. The intervention should be repeated at regular intervals, considering emerging needs and increasing awareness of families over time. Involving children and adolescents in advance care planning remains a challenge.

**Conclusion:**

A paediatric advance care planning intervention should take into account potential pitfalls and barriers including issues related to timing, potential conflicts between parents and professionals, ambiguity towards written advance directives, the role of non-medical carers for paediatric advance care planning implementation, the need to involve the child and the necessity of an iterative process.

**What is already known about the topic?**

- Advance care planning comprises discussions with patients, surrogates and healthcare providers about preferences for future treatment and care considering goals, values and beliefs of patients.
- Advance care planning concepts for adults are not directly applicable to the paediatric setting.
- Existing models of paediatric advance care planning tend to target special groups of patients or to be limited to single elements of the process like advance care discussions or advance directives. A common understanding of paediatric advance care planning is still missing.

**What this paper adds?**

- As part of an overarching project aimed at developing a paediatric advance care planning intervention, we report about the requirements and key components of paediatric advance care planning identified in transdisciplinary workshops.
- Results integrate the perspectives of bereaved parents, healthcare providers and stakeholders of care networks to ensure a comprehensive understanding of paediatric advance care planning including discussions, documentation and implementation.
- This paper particularly contributes to the knowledge about timing issues in paediatric advance care planning, including the need for an iterative process, barriers to paediatric advance care planning implementation and the necessity to enable non-medical stakeholders in care networks to ensure care in accordance with families’ preferences. So far, research has paid little attention to this issue.

**Implications for practice, theory or policy**

- Results will inform the fundament for the development of a comprehensive paediatric advance care planning intervention that will be evaluated in a pilot study.

## Introduction

Advance care planning is a structured and evidence-based model that enables individuals to determine goals and preferences for future medical treatment and care.^1^ It includes professionally facilitated discussions between a person, his or her close relatives and healthcare providers, a valid documentation of preferences and anticipatory decisions, and their effective communication and implementation.^2,3^

International guidelines and medical societies recommend that families and healthcare providers have early discussions about goals of care and treatment options in paediatrics.^4–9^ However, to our knowledge, there are still few structured models of paediatric advance care planning worldwide.^4,10–15^ Existing approaches tend to be limited to single elements of the process (e.g. advance directives) or targeted at special groups of patients (e.g. adolescents with a life-shortening condition). A common understanding of paediatric advance care planning is still missing. Another widely neglected issue concerns respecting advance directives outside hospitals.^16–18^

Previous research has shown that adult advance care planning concepts are not directly applicable to paediatric situations.^6,10,19,20^ Advance care planning in paediatrics mostly concerns children with complex medical conditions, long and variable disease trajectories and uncertain prognoses. Many children do not have the capacity to consent due to developmental immaturity or communication impairment. Thus, paediatric advance care planning typically involves surrogate decision-making by parents. Further challenges are high emotional impact of end-of-life decisions for children, involvement of large care networks and uncertain legal status of advance directives.^4,19–23^ Therefore, a specific paediatric advance care planning intervention tailored to the needs of parents, patients and professionals is needed.^4–6,9,10,24–28^

Previous studies suggest that paediatric advance care planning increases sense of control, provides peace of mind, reduces suffering and supports families in coping with their situation.^5,10,29^ However, professionals feel insecure about when and how to start paediatric advance care planning discussions.^19,29,30^ They are concerned about the lacking legal validity of advance directives for children^10,19^ and worried about burdening families and destroying their hopes and their trust in physicians.^5,19,29^ Some parents do not feel ready to engage in paediatric advance care planning and show ambivalent feelings towards end-of-life conversations.^5,10,19^ As the first step of a comprehensive project aimed at developing a paediatric advance care planning intervention, we strove to identify requirements and key components of paediatric advance care planning taking into account the needs of all involved parties including parents, healthcare providers and stakeholders of care networks.

## Methods

### Design

The purpose of participatory research is to include study participants as equal partners in the research process.^31,32^ Accordingly, we invited participants to constitute an advisory board that accompanies the whole paediatric advance care planning project and organised two transdisciplinary workshops^33^ at the Centre for Paediatric Palliative Care in Munich. The first workshop (1 day) took place in March 2018, the second (2 days) in July 2018.

The project was accompanied by a scientific advisory panel with national and international experts in paediatric palliative care. The scientific advisory panel was gender balanced and comprised three physicians (MD) – two experts in palliative care and paediatrics, one expert in ethics – two nurses (RN, PhD) and one social worker (MA, PhD).

The Munich University Hospital Ethics Committee approved the study protocol and materials in February 2018 (project no.: 17-885). The methods employed for the study and its reporting follow the consolidated criteria for reporting qualitative research (COREQ).

### Sampling and recruitment

Qualitative research strives for transferability of results, which does not depend on the number but on the type of participants involved in the study.^34–36^ Eligible participants were considered as follows: (1) health care professionals of different professions and healthcare institutions in Bavaria, who are regularly involved in the care of children with life-limiting diseases and will themselves conduct discussions about treatment limitation and end-of-life care (facilitators); (2) stakeholders of the care network who regularly face written documents without having been involved in advance care discussions (implementators); and (3) bereaved parents whose children had been cared for by the paediatric palliative care team in Munich. We assumed that these parents were in a better condition to talk about sensitive issues due to temporal distance and a pre-existing relation of trust with professionals of the centre. We did not include parents of current patients since they might be in the stressful process of decision-making for their own child and this could interfere with their participation in the research. Parents of current patients could also feel inhibited in the presence of treating physicians during workshops. In this study, we focused on the parents as surrogate decision makers; thus, we did not include young patients as participants in the discussion groups. All participants were informed about the study and gave their written consent.

### Data collection

At the first workshop, participants were assigned to one of three discussion groups according to their background: (1) parents, (2) facilitators and (3) implementators, with the aim to explore their experiences with paediatric advance care planning (Table 1). During the second workshop, we invited participants to choose two out of four mixed dialogue groups on the following issues: participation of children and adolescents, paediatric advance care planning documentation, implementation and supplementary written materials (Table 2). The topics of the dialogue groups emerged during the analysis of discussion groups in the first workshop. Dialogue groups were inspired by a method called ‘Victorian Calling’.^37^ Participants were free to choose their topics of interest and received the points of discussion in advance. Preparation of the guidelines for discussion and dialogue groups was discussed with the Munich working group on qualitative methodology and the study’s scientific advisory panel.

**Table 1.** Participants and guidelines of discussion groups during the first transdisciplinary workshop.

Parents	Facilitation	Implementation
5 mothers 2 fathers	4 paediatricians 1 psychologist 1 chaplain 1 nurse (intensive care)	1 emergency physician 2 nurses (outpatient) 2 social workers 3 pedagogues
– Which experiences have you had with any form of advance care planning? – How did you perceive the process of decision-making? – How was the relationship and communication among the participants in the pACP discussions? – Which experiences have you had with written pACP documents such as advance directives? – What would you recommend for other parents and professionals concerning pACP? – Under which circumstances did you find it not helpful or even harmful to have pACP conversations?	– Within your work context, how was your experience with any form of advance care planning or advance directives in children? – Did you conduct or participate in pACP or similar conversations? Can you tell us about your experience? – Did you use any kind of pACP documentation or advance directives? Can you tell us about your experience? – What would be your recommendations regarding pACP?	– Within your work context, how was your experience with any form of ACP or ADs in children? – Have you ever been faced with pACP decisions, pACP documents or an AD in children? Can you tell us about your experience? – How did your institution handle the ADs or the pACP documents? – What are your recommendations for the documentation of the pACP process and the pACP and/or ADs?

pACP: paediatric advance care planning; ACP; advance care planning; AD: advance directive.

**Table 2.** Topics of dialogue groups (Victorian Calling).

Dialogue groups	Topics
Participation of children and adolescents	Timing, format, information material, family discussions and bilateral talks, divergent wishes, information of other family members, documentation for children/adolescents
Implementation	Facilitators, barriers, special requirements, information about process, information about content, format of communication, possible implementators, contact persons, structural needs
Written information materials for parents	Which kind of information beforehand and which information during the process, way and timing of delivering, place of storage, person responsible for handing information over
Documentation of the pACP process	Documents needed, who needs what, which aspects/situations have to be covered, layout of documents, standard document, how to improve reliability and acceptance, timing of updates, what should not be included

pACP: paediatric advance care planning.

### Data analysis

Discussion and dialogue groups were audiotaped and transcribed verbatim. We deleted direct personal data completely and irretrievably from all transcripts. We further modified indirect personal data (e.g. names of places) in a way that allows data analysis but makes personal identification impossible. Data could only be accessed by the research team. Audios were deleted after concluding the study.

Transcripts of discussion and dialogue groups were analysed by content analysis according to Kuckartz^38^ and supported by the software MAXQDA 12. The team of coders was composed by two psychologists (K.H. and V.Z.) and one sociologist (D.R.). K.H. has a large experience in qualitative research and teaching of qualitative methods. D.R. and V.Z. were trained in qualitative methods before data collection and coding. We performed a descriptive, content-based analysis following a data-driven strategy. To this end, we used open coding^39^ to create a first list of codes. Codes were organised in a hierarchical coding frame with main categories and subcategories. The coding frame was tested in a second revision of the material and modified to fit data and reach consensus among coders. The resulting coding scheme was applied to the whole material including discussion and dialogue groups. In addition, we compared utterances of parents and professionals to identify group patterns and contrasting perspectives. The coding scheme is available on demand.

## Results

We included nine bereaved parents – six mothers and three fathers – of children of different ages (2–16 years), with diverse diagnoses across all together for short lives (TfSL) categories for life-limiting conditions (three metabolic, two oncological, two perinatal, one cardiological, one neuromuscular) in the advisory board. Two mothers and one father could not participate in both workshops, and one mother in the second workshop due to sickness, family celebrations or work-related reasons. In total, 14 healthcare providers and stakeholders participated in each workshop: 4 paediatricians, 1 emergency physician, 1 psychologist, 1 chaplain, 3 nurses (intensive care, outpatient), 2 social workers and 2 special education teachers. A vast majority of professionals were women (12 out of 14 during the first and 11 out of 14 during the second workshop). Two physicians could not attend the first, but participated in the second workshop. Two other physicians and one social worker could not attend the second workshop. Absent professionals did not obtain permission from their employers to attend the workshops. All parents (including the absentees) and all professionals but one expressed interest in continuing their participation in the advisory board. Data analysis highlighted five key elements of paediatric advance care planning: *decision-making discussions, documentation, implementation, timing and participation of children and adolescents* (the corresponding quotes can be found in an extended Table available online as Supplementary Material).

### Decision-making discussions

In order to reach a ‘right’ decision for their child, parents engaged in several decision-making discussions with the paediatric palliative care team, their partners, external healthcare providers and other persons of trust. Parents perceived decision-making as an ongoing communication process that transcended the conversations with paediatric palliative care professionals.

Unfortunately, these parental deliberations were hardly visible for the healthcare providers. Professionals thought that parents were reluctant to engage in decision-making discussions or too overburdened to make a ‘right’ decision. Some had the impression that parents would take sudden and inexplicable decisions.

Parents found it helpful to have several paediatric advance care planning meetings with facilitators. They asked that professionals take into account individual needs, place the focus on the child, discuss hypothetical scenarios and allow decision-making without pressure. Parents disapproved of insensitive communication, discussions at wrong times and places, unsuitable coping with emotions and lack of experience or knowledge on the part of professionals.

Parents and professionals endorsed discussions to be conducted in tandem by a clinician and a psychosocial professional or nurse. Participants expected clinicians to take the lead. They suggested that professionals contact them a few days after a meeting and ask if there were further questions or comments.

### Documentation

Participants discussed different types of documentation: the documentation of decisions (emergency recommendations, advance directives) and accompanying documentation of the process (minutes of discussions, journal). Professionals were more concerned about the documentation of decisions in order to have guidelines for future treatment. Parents were more focused on the decision-making process as such.

Professionals recommended the use of brief recommendations for emergencies, supplemented by larger advance directives containing a characterisation of the child, the diagnosis and the course of the disease. All participants agreed that all parties involved should sign the documents. Contact information should be easily retrievable and organised in accordance to priority. Professionals worried about the unclear legal status of advance care planning documents for children.

All participants recommended keeping minutes of all discussions to ensure continuity of the process. Other accompanying materials (e.g. leaflets about the programme or journals for parents and children) were seen as optional. Participants did not approve for supplementary written materials to be handed out without a personal conversation.

### Implementation

According to healthcare professionals, advance directives or emergency instructions need to be distributed among relevant stakeholders of the child’s care network, such as family and friends, the paediatrician in charge, home care nurses, teachers and in some cases even transport services. The original documents should be kept in proximity of the child and stored in reachable place. Copies of written documents should be signed, dated and tracked and data security provided.

Stakeholders wanted to receive and be informed about the documents in a personal conversation, in order to ask questions, to discuss emergency procedures and to address in advance potential conflicts between institutional policies and the family’s wishes. Stakeholders from educational and care facilities proposed to designate responsible persons for emergencies, to participate in case-specific round tables with all parties involved, to have regular trainings and to support institutional networking. Home-based nurses wished to participate in the paediatric advance care planning process to share their perspective on the child.

Healthcare providers and stakeholders reported several barriers to providing care consistent with the families’ preferences:

First, stakeholders of educational and care facilities informed about disagreements with parents on end-of-life decisions for the child that caused them high levels of moral distress. Parents, on the other hand, felt burdened when professionals challenged their decisions or questioned existing documents.

Second, in case of emergency, staff members of educational and care facilities had major difficulties to stay with a dying child and to cope with the emotional strain without attempting resuscitation or calling emergency services. In their own words, they could barely ‘endure doing nothing’.

Third, stakeholders emphasised the need for social support, arguing that lone professionals are likely to feel overwhelmed and to proceed against a parental wish to forgo resuscitation.

Fourth, professionals were concerned about a sudden change of mind of parents and thus felt unsure about applying existing advance directives during a crisis. Clinicians required a final validation of the documents by parents in the concrete situation before acting in conformity to them.

Finally, some emergency physicians may not respect parental choices due to legal uncertainty, lack of knowledge about the child and little experience with rare diseases and paediatric palliative care.

To prevent barriers to implementation, participants proposed to inform and train relevant stakeholders, to maintain a personal contact with them and to involve the paediatric palliative care team in case of conflict or during an emergency.

### Timing

#### The right time to start

Professionals were concerned about the possible lack of readiness of parents to engage in paediatric advance care planning. According to professionals, when parents are not ready, they are more likely to reject treatment limitations for their child and less likely to participate in paediatric advance care planning discussions or to complete advance directives.

Parents confirmed that there was a time during which they preferred to avoid thinking about end-of-life issues. However, at some point, they realised that their child was not going to get better. Parents described this moment as a turning point, after which they felt ready to engage in advance care planning.

Although most participants favoured an early start of paediatric advance care planning, some parents questioned this approach and demanded a previous assessment of parental readiness. However, even bereaved parents were not able to give a clear definition of a ‘right time’ to initiate advance care planning. On the other hand, they described in detail what they considered as wrong times: shortly after breaking bad news, shortly after overcoming a crisis or under time pressure.

All participants indicated that ‘timing might never be right’. However, missed opportunities to engage in paediatric advance care planning may lead to regrets. One solution might be to offer families timely to participate in paediatric advance care planning and to repeat this offer regularly in case parents do not feel ready.

#### Iterative process

Participants conceived paediatric advance care planning as an iterative process suggesting re-initiating the process every 3–12 months, according to the individual case. Parents may not be aware of the necessity of updating documents; thus, professionals should take the initiative and guide parents through process iteration.

#### Sequential steps

Participants recommended embedding paediatric advance care planning in the continuous care of families. Care should start as soon as possible and respond to the emerging needs and increasing awareness and acceptance of the situation during the course of the disease. Participants used the metaphor of ‘putting pearls on a string’ to describe this step-by-step approach.

### Participation of children and adolescents

Professionals regarded the participation of children of all ages in paediatric advance care planning as self-evident whereas parents were sceptical about involving young children. Parents worried about healthcare providers being insensitive and scaring younger children off. However, both agreed that concerned adolescents should be offered separate conversations with professionals.

Some professionals complained about parents acting as gatekeepers preventing them to talk to children. They wanted to obtain support in talking with parents about their child’s participation in paediatric advance care planning. On the other hand, parents asked for support to be able to talk themselves about sensitive issues with their children. We also identified a latent conflict between parents and institutional care workers, both claiming to be experts and advocates for the child.

## Discussion

### Main findings

This paper reports about key components of paediatric advance care planning and the requirements for its documentation and implementation identified through a qualitative, participative approach including bereaved parents, health care providers and stakeholders of care networks. Participants understood paediatric advance care planning as an iterative, communication-based decision-making process. This is consistent with the literature on shared decision-making, which conceives advance care planning as a collaborative effort that allows patients, surrogates and clinicians to make healthcare decisions together, taking into account scientific evidence as well as the patients’ values, goals and preferences.^40^ Consistent with prior research, parents focussed on decision-making discussions and paid little attention to written documents.^5^ Notably, a major part of decision-making takes place outside scheduled meetings and is thus invisible to healthcare providers unless they maintain an ongoing contact with parents.

Healthcare professionals, on the other hand, showed a high interest in written documents. Until now, there are no standard and legally binding advance directives for minors in Germany.^22,23^ Thus, there is a necessity to develop a clear documentation of decisions to ensure that all stakeholders of the care network are timely and correctly informed about care preferences for children.^5^ A recent study about advance directives for children in Germany indicates that professionals would like to have a concise decisional overview for use during emergencies as well as a detailed informative documentation.^22^ Our participants’ suggestion to have brief recommendations for emergencies supplemented by larger advance directives containing detailed information about the child mirrors these findings.

Previous studies have rarely discussed the implementation of paediatric advance care planning outside the hospital, or looked at the specific needs of stakeholders such as personnel in educational institutions (e.g. kindergarten, school, day care), who are major gatekeepers in the quest for respect of parental choices.^10,41^ Among the main hindrances to the provision of care consistent with the preferences of families were disagreements between parents and professionals, difficulties with emergency services and emotional barriers of stakeholders including the refusal of forgoing resuscitation due to emotional strain and missing professional support during emergencies. Kimberly et al.^16^ conducted a study on the implementation of paediatric advance care planning documents in schools, finding that most US institutions do not have a policy or procedure regarding these documents. Teachers in United States also have great psychological barriers when it comes to forgoing resuscitation, and families have difficulties finding nurses willing to honour such decisions. Non-resuscitation of children was interpreted as a violation of the duty of protection.^17,42^

Barriers to implementation remind us that paediatric advance care planning is more than just having advance care discussions and completing advance directives. We need to find a way to involve relevant stakeholders of the care networks in order to enable them to provide care in accordance with the preferences of families. Possible strategies to this end mentioned by the participants included maintaining personal contact with the paediatric palliative care team, exchanging information with other involved stakeholders in round tables and participating in regular trainings on paediatric palliative care. Further research on the implementation of paediatric advance care planning in the community appears warranted.

Other relevant findings of our study concern the issue of participation of children and adolescents in paediatric advance care planning. Current debates demand children and adolescents with a life-limiting condition to be part of decision-making discussions.^7,13,43–45^ However, these studies are usually limited to the participation of communicative and competent teenagers, and tend to disregard the fact that the majority of paediatric palliative care patients have severe cognitive impairments or are too young to participate in decision-making discussions. Our findings highlight the potential for conflicts between parents and professionals about the child’s best interests. These diverging perspectives need to be addressed by future research, as they may become an important barrier to the implementation of paediatric advance care planning.

The right time to initiate paediatric advance care planning constitutes a major concern for practitioners.^19,29,30^ Most investigations recommend initiating advance care planning as soon as possible.^29,46–48^ Only a few studies express concern about this recommendation.^5,49^ Our findings suggest that it is of paramount importance to take parental readiness into account and to avoid wrong timing of conversations. However, our results do not point to a clear strategy for assessing parental readiness. A possible solution might be to integrate paediatric advance care planning in the continuous care of families. Teenagers with cancer supported having end-of-life discussions well in advance of the dying phase.^50^ Professionals should offer discussions at regular time intervals to avoid missing opportunities.

Overall, participants envisioned paediatric advance care planning as a comprehensive intervention involving repeated rounds of conversation embedded in an ongoing support with a sequential approach. These results are consistent with previous research.^19,22,49^

### Strengths/weaknesses

We used a participatory approach to ensure an active involvement of participants and enable them to co-determine the design of the study. Development of the intervention followed a bottom-up strategy instead of adapting adult advance care planning to paediatrics,^11,14,51^ in order to ensure that the programme fits to the specific needs of paediatric palliative care patients, families, healthcare providers and concerned stakeholders. The diversity of participants enabled us to cover the whole process of paediatric advance care planning including discussions, written documents and their implementation.

Our recruitment strategy entails several restrictions that limit the capacity to transfer our findings to other contexts and subjects: we only recruited professionals in Bavaria and bereaved parents at the Centre for Paediatric Palliative Care in Munich. We also excluded parents of current patients in paediatric palliative care and did not include children or adolescents in the sample; thus, their perspective is missing. We also had missing attendees during both workshops. Overall, it is difficult to estimate the influence of non-attendance on the interpretation of data as discussion and dialogue groups depend on group dynamics rather than the point of view of single individuals. However, parents were present and active in both the first and second workshop.

### What the study adds

This paper particularly contributes to the knowledge about timing issues in paediatric advance care planning, including the need for an iterative process taking into account parental readiness; barriers to paediatric advance care planning implementation, including the ambivalence of medical professionals towards parental advance directives; and particularly, the necessity to involve non-medical stakeholders in care networks in the paediatric advance care planning process in order to enable them to provide care in accordance with the families’ preferences. So far, research has paid little attention to this issue.

## Conclusion and outlook

Our qualitative study identified elements of paediatric advance care planning considered as crucial from the perspectives of parents, healthcare providers and stakeholders of care networks. We reported these results back to the members of the advisory board and asked them to use this information to help us develop a comprehensive paediatric advance care planning intervention. We are currently evaluating the feasibility and acceptability of the developed paediatric advance care planning concept in order to establish a local best practice. Adaptation and dissemination of the paediatric advance care planning (ACP) concept to other paediatric specialties and paediatric palliative care teams in Bavaria and Germany are the next planned steps.

## Supplemental Material

Hein_et_al._Online_Suppl._Material_Table_with_quotes_end – Supplemental material for Identifying key elements for paediatric advance care planning with parents, healthcare providers and stakeholders: A qualitative study Click here for additional data file. Supplemental material, Hein_et_al._Online_Suppl._Material_Table_with_quotes_end for Identifying key elements for paediatric advance care planning with parents, healthcare providers and stakeholders: A qualitative study by Kerstin Hein, Kathrin Knochel, Vedrana Zaimovic, Daniel Reimann, Anna Monz, Nari Heitkamp, Gian Domenico Borasio and Monika Führer in Palliative Medicine
