# Supplementary material for: Identifying key elements for paediatric advance care planning with parents, healthcare providers and stakeholders: A qualitative study
Source: Palliat Med. 2020 Jan 27;34(3):300–8. doi: 10.1177/0269216319900317 (PMC7074656; doi:10.1177/0269216319900317)
Supplement: Hein_et_al._Online_Suppl._Material_Table_with_quotes_end – Supplemental material for Identifying key elements for paediatric advance care planning with parents, healthcare providers and stakeholders: A qualitative study [file Hein_et_al._Online_Suppl._Material_Table_with_quotes_end.pdf]

Table. Key elements of pACP: Themes and illustrative quotes

Key elements	***Themes***	***Sub-themes***	***Illustrative quotes***
DECISION-MAKING DISCUSSIONS	Parental perspective	• Ongoing communication process<br>• Different dialogue partners: palliative care team, partner and significant others	“Simply because it is good. Talking to others and listening to their opinion clarifies the thoughts in your head” – Parent, discussion group, ongoing communication process<br><br>“During the course of time we always had about three whom we trusted and who came from different areas. One of them was a paediatrician, the other an oncologist from another hospital, and just now the current attending physician of the palliative care team” – Parent, discussion group, different dialogue partners
	Professionals’ perspective	• Parental deliberation is not visible for professionals	„Yet they [parents] do not want a written commitment and they do not want do engage in discussions. And then, at some point, it comes as it comes.” – Facilitator, discussion group, parents avoiding decisions
	Facilitating factors	• Several meetings<br>• Time during and between meetings<br>• Multi-professional team<br>• Focus on the child<br>• Discussing hypothetical scenarios<br>• Non-judgemental discussions<br>• Feedback loop	„You just have time. You don’t have to decide everything overnight. To know this was incredibly reassuring for us” – Parent, discussion group, time during and between meetings<br><br>“I also think that it is important to remain open about the results of discussions. Things are not clear. It should not be only about doing what the physician says.” – Parent, discussion group, non-judgemental discussions<br><br>„There are many children who are not capable to express their needs. As parents, we should not only do what we think is the best or what seems to be best for the family. We first have to find out the will of our children and then we have to write it down somehow […] And I think this is very important. This should be a main component of such discussions. We should find out: Who is my child? What kind of child is he? How is he doing? What does he need?” – Parent, discussion group, focus on the child<br><br>“This has accompanied us very well. To discuss from the outset: what may happen? What stages lie ahead of us? To try to imagine these situations at least to some extent and to develop an inner attitude towards them. What will these stages do with us? How do we want or how can we integrate these situations in our lives? What will be our way?” – Parent, discussion group, hypothetical scenarios

			"And this would be, I think, quite good, if you have such a little feedback-talk simply a few days later on" – Parent, discussion group, feedback loop
	Barriers	• Insensitive communication<br>• Discussions at wrong times and places<br>• Unsuitable coping with emotions<br>• Professionals lacking experience or knowledge about the child's disease<br>• Diverging perspectives between parents and professionals	„They [physicians] actually talk about it like they talk about opening a bank account (laughter). So he told us about all the changes that will happen in our lives in about one and a half, two hours, how our entire life will change […] I already stopped listening after 10 minutes" – Parent, insensitive communication<br><br>"We were on rehab, which was nice for the whole family […] Our daughter was doing fine. We just felt that she managed to survive once more and that everything was now fine. We could go back to normality. Then she [physician] asked us, well, have you ever thought about death? (laughter) How do you want to prepare yourself? Certainly, there was the possibility of a relapse. Of course, she was right […] However, at that time, we didn´t want to think about it. We were living in the present […] So, I was totally shocked" – Parent, discussion group, discussions at wrong times and places<br><br>"So, at the first encounter, the physician started the conversation with: 'Actually, I don't know exactly how to do this. This is my first time' (laughter, overall chatter) I really understand it from a human point of view. But this didn't really inspire much confidence" – Parent, discussion group, lacking experience of professional<br><br>"We had the experience that the palliative care team did not want what we wanted. Then we had to go to other physicians who gave us what we wanted. […] For us, it was important to prolong life. The palliative care team did not want to prolong life. But we wanted our daughter to enter school, because this was her greatest wish. And this was only possible with another chemo. The palliative care team refused the chemo, because they did not want to support life-prolonging measures. So we got the chemo from the paediatric cancer ward. However, this was a major breach of trust." Parent, discussion group, diverging perspectives
DOCUMENTATION	Resulting documentation	• Emergency recommendations<br>• Advance directives	„Then we have the key decisions. What does the emergency physician have to know in the first place? Followed by the plan on how to manage a crisis […] this should also be documented" – Facilitator, dialogue groups, emergency recommendations<br><br>"Once we have managed a crisis and are in intensive care with an intubated child […], how far do we go with treatment? […] in that situation, it would be helpful simply to understand, to have a written document after having had some kind of

			discussions. As a treating physician, I would like to read this document, to be able to say, okay, these are the values of the family, this is their spiritual context […] This is preparatory work for treating professionals, to be able to make a good decision for the child in a shorter time span. On the long-term, this means to make a good decision for those left behind, parents and siblings" – Facilitator, discussion group, advance directive
	Accompanying documentation	• Minutes of discussions<br>• Journal for parents and patients	„At least in our case, I see the problem that we don't take minutes to make discussions transparent for everybody. Who has conducted which discussion, when and about what? Is there a treatment plan so we do not have to discuss this all over again at each handoff? Is there a clear guideline, to which we can adhere and that we all can or have to live with?" – Facilitator, discussion group, minutes of discussion<br><br>„What kind of information would we need during this process […] that parents somehow have the opportunity […] to recapitulate in a written form, meaning to write down what they are thinking or what they still need, so they can reflect about it by taking notes" – Facilitator, dialogue group, journal for parents and patients
	Supplementary information material	• To be handed out only after personal conversation	„Discussions should always be conducted. I think, you cannot simply hand out the flyer and tell the person, just read it." – Facilitator, dialogue group, supplementary materials
IMPLEMENTATION	Barriers to provide care consistent with preferences	• Disagreements between parents and professionals –enduring parental decisions<br>• High emotional strain caused by forgoing resuscitation of children<br>• High emotional strain due to missing professional support during a crisis<br>• Concerns about parents suddenly changing their mind during a crisis<br>• Emergency physicians with little knowledge about the child, the underlying disease, and principles of palliative care<br>• Legal uncertainty around pACP documents for children	"We, as nursing service, are sometimes so powerless, that, at one point, we have to say to ourselves, when all offers are exhausted, that we then say, it´s the parents' child and we are only guests and we have to endure this" – Implementator, discussion group, enduring parental decisions.<br><br>"Then we had a nurse from the nursing team who was not willing to accept palliative care. We were not talking about immediate end-of-life care. We just wanted to avoid connecting our son to all sorts of available machines. We wanted to maintain our son's quality of life for as long as possible without prolonging the suffering. This might sound a little bit pathetic, but somehow, that was our formula. And she acted against our wishes on several occasions, until we said that we did not want her anymore." Parent, discussion group, disagreements between parents and professionals<br><br>"But now following a piece of paper and to say, yes, it is written here, resuscitation is not wanted – this is impossible. Someone has to have been through this, this

			situation, to be able to emotionally understand it. It is brutal. It happened in October and it still haunts us" – Implementator, discussion group, high emotional strain caused by forgoing resuscitation of children.<br><br>"This okay, this nod, this blessing by the parents or by the mother, it doesn´t matter, whoever is there, is totally important, in my opinion" – Implementator, discussion group, concerns about parents suddenly changing their minds.
	Facilitating factors	• Personal conversation or round table to inform all care persons involved<br>• Designate responsible persons for emergencies<br>• Regular training of stakeholders in care networks<br>• Cooperation with paediatric palliative care team	"Exactly, I think that we have to organize at least one big round table, during which everybody presents themselves and their positions […] I think it is important to have the palliative care team, the paediatrician […] Also important are the school, the kindergarten, the nursing service. And then we have to reduce it, later on, when you are with the family. Then it is the mother, the father, the nurses, the palliative care team." – Implementator, dialogue group, round tables<br><br>"At that moment [emergency situation], there should be one head giving the directives. It should become increasingly sharper" – Implementator, dialogue group, designate responsible person<br><br>„Regarding professionals, for me it is important that all involved professionals receive the corresponding information. This means that they should participate in trainings about pACP such as this one" – Implementator, dialogue group, regular trainings
TIMING	The right time to start	• As soon as possible<br>• Consideration of parental readiness	"I think that it always is a long lasting process, I think, a lot people forget that. We often have this attitude, yes, palliative care, in, right away, quickly in. But the parents are not ready yet. And I think, one should not rush things, because they will block you out" – Implementator, discussion group, parental readiness
	Iterative process	• Repetition of discussions at regular time intervals<br>• Update documents at regular time intervals	"Until now, we had a sort of matrix that considered three, six or twelve months. From our point of view, these are the points in time, at which we will certainly need an update to avoid missing something. That's what we thought." – Facilitator, dialogue group, update at regular time intervals
	Sequential steps	• Discussions embedded in continuous care of the families<br>• Consideration of emerging needs of the families during the course of the disease<br>• Consideration of increasing awareness and	"Well, I really think that we do our best when we work in sequential steps. This means that we do not focus on precise topics within the discussions. Instead, a) we know that roles are distributed and b) we know that we can always add something new to the process, like pearls on a string. At best, we will trigger a slow development towards acceptance, although parents may express several ambivalences until the end. This is indeed a difficult topic" – Facilitator, discussion

		acceptance of the situation during the course of the disease	group, sequential steps<br><br>"It would be too early at that time, if we say, oh, let's think about the time when he needs ventilation. Of course, this is nonsense because you may still have ten years until then […] And then it may happen that discussions continue one year later because your child has difficulties walking […] But still, it would be too early to think about difficulties swallowing, about aspiration issues or ventilation […] But then, we would like to know about these issues when we reach a stage at which things become more complicated. As you said, this is the point when it may happen at any time. Then, aspiration may become medically relevant and parents should have the necessary technical devices it at home […] The discussion should prepare for these situations, always related to the course of the disease." – Parent, dialogue group, sequential steps
PARTICIPATION OF CHILDREN AND ADOLESCENTS	Professionals' perspective	• Participation is self-evident<br>• Parents act as gate-keepers<br>• Need of support in talking with parents about child's participation	"I think that it is not a question about whether you involve children and adolescents, but rather a question about how to do it" – Facilitator, dialogue group, participation is self-evident<br><br>"And we also have families that, really from start to death, do not explain to their children that they are sick, and also forbid us to talk to the child […] And then we stay at the bedside and almost despair" – Implementator, dialogue group, parents as gate-keepers<br><br>"How can I approach parents? How can I inform parents that it is important that even children aged two or three are guided into these conversations?"– Facilitator, dialogue group, support in talking with parents
	Parental perspective	• Scepticism towards participation of young children<br>• Need of support in talking with children	"Okay, I imagine a child. Let's say five, six years old. And I have this child sitting in front of me. And now I discuss with him, if he wants to have a gastric tube or these sorts of things, which I believe, this is too much for the child. Thus, I would say I would completely refuse it for children. But for adolescents, yes." – Parent, dialogue group, skepticism towards participation of young children<br><br>"When we asked how to do it, they said, we know you can do it. Just find a way to tell it to your child. Now, go and tell a seven-year-old child that he is sick and that the disease implies this and that. Thus, they left us quite alone. At that time, it was like that for us. A guidance would have been a good thing" – Parent, dialogue group, need of support in talking with children

	Who is the expert for the child?	• Parents and professionals claim to be experts on the child	“We really look at it from the child’s perspective. And we experience the child in a totally different setting, something the parents don´t. Well, they are always totally surprised, okay, what their child can do and how it reacts and so on. And I believe, this is also a very valuable perspective of our staff, who have such a close relationship with the child – same as the nurses – to simply have another representation of the child.” – Implementator, discussion group, experts on the child
